# Steatosis and hepatitis C

**DOI:** 10.1093/gastro/gov040

**Published:** 2015-08-13

**Authors:** Jamak Modaresi Esfeh, Kianoush Ansari-Gilani

**Affiliations:** ^1^Department of Gastroenterology and Hepatology, The Cleveland Clinic, Cleveland, OH, USA; ^2^Department of Radiology, University Hospitals Case Medical Center, Case Western Reserve University, Cleveland, OH, USA

## Abstract

Hepatitis C virus (HCV) infection is a common liver disease worldwide with a high rate of chronicity (75–80%) in infected individuals. The chronic form of HCV leads to steatosis, cirrhosis and hepatocellualr carcinoma. Steatosis is prevalent in HCV patients (55%) due to a combination of viral factors (effect of viral proteins on some of the intracellular pathways) and host factors (overweight, insulin resistance, diabetes mellitus, and alcohol consumption). The response rates to treatment of chronic HCV with pegylated interferon (PEG-IFN) and (in the case of genotype-1 HCV, the most common infecting genotype in the USA) ribavirin (RBV) is low, with a sustained viral response rate ≤ 40%. Adding direct-acting antiviral agents—recently approved by the FDA—to the standard protocol has increased the response rate; however HCV-related end-stage liver disease is still the primary indication for liver transplantation in the USA. The focus of this article is on the interrelation between HCV, steatosis and metabolic syndrome.

## Introduction

Hepatitis C virus (HCV) is a global health problem, affecting about 180 million individuals. It has been estimated that 3.9 million Americans are positive for anti-HCV antibodies. Of these individuals, 74% also had positive HCV RNA test results, indicating that approximately 1.33% of the population of the USA (that is, 2.7 million people) have chronic hepatitis C infection [1].

**Table 1.** Predictors of an unfavorable response to treatment with peginterferon and ribavirin

**Viral Factors**
High pre-treatment HCV RNA load
(genotype 1)
**Host Factors**
Unfavorable IL-28B genotype (e.g. T/T)
African American ethnicity
Male gender
Age > 40 years
Obesity
Steatosis
Insulin resistance
Presence of liver fibrosis
HIV co-infection
Non-compliance with the treatment

Hepatitis C is also the most common chronic blood-borne infectious disease. The most common mode of HCV transmission is through blood transfusions, hemodialysis, and organ transplants, but the sharing of used needles and razor blades, and the use of tattooing guns have also contributed significantly to the spread of the virus [2].

**Figure 1.**
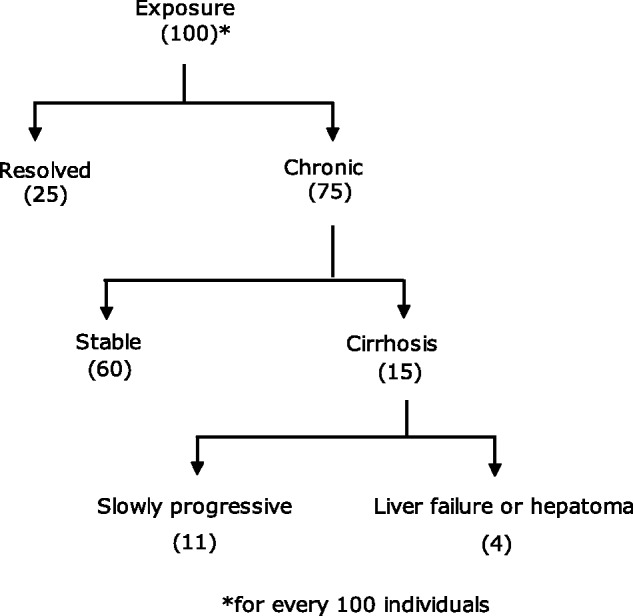
Natural history of hepatitis C viral infection.

It is the chronicity of hepatitis C and its complications which makes it troublesome; it has been estimated that 75–80% of individuals infected with HCV progress to chronic infection, persisting for at least 6 months after onset, with the rate of chronic infection varying by age, sex, race, and immune system status. Long-term infection has been associated with serious clinical sequels, including the development of hepatic fibrosis, cirrhosis and hepatocellular carcinoma (HCC). Although the natural history of HCV infection is believed to be very variable, it has been estimated that up to 15% of chronically infected individuals will develop cirrhosis of the liver over a 20–25 year period and that these individuals are at increased risk for developing end-stage liver disease or HCC. It has also been estimated that, of patients with cirrhosis, approximately 4–5% will develop HCC each year [1]. The natural history of HCV infection is shown in Figure 1.

Metabolic defects, including obesity, diabetes mellitus (DM) and hyperlipidemia have recently emerged as potential co-factors in the development of complications in the setting of chronic HCV [3, 4].

## Steatosis

Hepatic steatosis is a generic term referring to lipid accumulation within hepatocytes [5]. Steatosis can be found in many liver diseases, namely non-alcoholic steatohepatitis (NASH) and hepatitis C virus infection [6]. There are several factors which can affect the development of steatosis in chronic hepatitis C: (i) viral factor (HCV genotype3), (ii) host factors (alcohol consumption, overweight, hyperlipidemia, diabetes mellitus, insulin resistance) and (iii) drug therapy (corticosteroids, amiodarone, methotrexate etc.) [7].

Although the mechanisms underlying the development of parenchymal steatosis in HCV infection are not exactly known, there are some findings to describe the mechanism of fat accumulation. The first series of mechanisms basically focuses on HCV proteins as the first step of the damage sequence. At least two HCV proteins (core protein and NS5A) are suspected to interact with the cell machinery involved in lipid metabolism; they inhibit microsomal triglyceride transfer protein (MTP) activity, which is a rate-limiting enzyme with a key role in the assembly of very low density lipoprotein (VLDL). The direct and likely consequence of this is accumulation of triglyceride in the cells causing steatosis [7, 8]. Others suggest that HCV core protein is capable of both inducing overproduction of reactive oxygen species (ROS) and attenuating some of the antioxidant systems, which may explain the mechanism underlying the production of a strong oxidative stress in HCV infection, compared with other forms of hepatitis. This is due to mitochondrial dysfunction [7–10]. Meanwhile it has been shown that, in healthy individuals, peroxisome proliferator-activated receptor alpha (PPAR-α) acts to ameliorate steatosis but, in the presence of mitochondria dysfunction—which can be seen in HCV patients—PPAR-α may exacerbate steatosis [8, 10].

**Figure 2.**
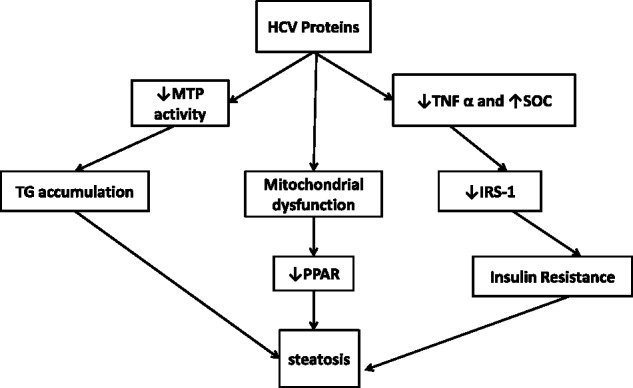
HCV-induced steatosis.IRS-1 = insulin receptor substrate-1; MTP = microsomal triglyceride transfer protein; PPAR-α = peroxisome proliferator-activated receptor alpha-α; SOCs = suppressor of cytokine signaling; TG = triglyceride; TNF α = tumor necrosis factor α.

The second proposed mechanism recognizes insulin resistance (IR) as the major mechanism in the pathogenesis of hepatic steatosis [12]. IR causes impaired metabolic clearance of glucose, compensatory hyperinsulinemia, and increased of lipolysis: the latter leads to increased plasma free fatty acids (FFAs), which will cause an increase in hepatic uptake of FFAs and finally results in steatosis [13]. Meanwhile, IR itself may result from excess FFAs, tumor necrosis factor alpha (TNF-α) and suppressor of cytokine signaling (SOCs), which could down-regulate insulin receptor substrate (IRS)-1 signaling [14, 15]. This will result in the impaired translocation of GLUT-4 transporters to the plasma membrane, which limits glucose uptake, increases blood glucose, and causes a compensatory increase in insulin [16]. Figure 2 shows a summary of the above mechanisms.

In chronic hepatitis C patients, the prevalence of steatosis ranges from 40–86% (mean 55%). The majority (78%) of patients with steatosis have mild steatosis affecting less than 30% of hepatocytes. In the western world, steatosis occurs more frequently in patients with chronic hepatitis C than in the general population of adults (in 55% of cases against 20–30%). Accumulation of fat has different pattern of distribution, depending on the underlying etiology; macrovesicular steatosis is founded in the periportal region of the liver, differently from the centrilobular distribution characteristics of NASH patients [6].

It has previously been shown that steatosis associated with HCV genotype 1 infection is a surrogate marker of metabolic abnormalities—similarly to what is seen in patients with NASH, including obesity, hyperlipidemia or DM—and has been termed “metabolic fat” to reflect this association. Interestingly, steatosis in patients with HCV genotype 3, while more common, is associated with viremia rather than metabolic abnormalities and has been termed “viral fat” where it may resolve after viral clearance [17].

It seems that HCV genotype can play a role in inducing steatosis; moderate or severe steatosis is significantly less frequent in genotype 4 than in genotype 3 and it is similar between genotypes 4 and 1. In non-diabetic, overweight patients, moderate or severe steatosis is present in only 10–15% of genotype 4 or 1 patients, compared with 40% in genotype 3. Thus, hepatic steatosis in genotype 4 is mostly associated with metabolic factors, similar to those in genotype 1[14]. Genotype 3 has the highest association with steatosis, which is present in 73% of patients with genotype 3 and in 50% of patients infected with other genotypes [7].

Steatosis has become an important issue in hepatitis C, due to the injury it can cause in a chronically ill liver [19], although a certain amount of lipid storage may even be hepatoprotective, but prolonged lipid storage can result in an activation of inflammatory reactions and loss of metabolic competence [21]. Steatosis in HCV patients is associated with more severe histological injury and higher fibrosis scores, suggesting that fat in the liver is a biologically active tissue. Steatosis can be significantly and independently associated with fibrosis in chronic hepatitis C. IR is the possible link between the metabolic abnormalities in HCV patients with steatosis to progressive hepatic fibrosis. Hyperinsulinemia in HCV patients may potentially promote fibrogenesis through either altered cytokine production—including TNFA—or by its direct effect on hepatic stellate cells [19, 22].

Resistin is an adipocyte-secreted hormone that belongs to the family of cystin-rich resistin-like molecules. This hormone antagonizes insulin action in rodents but its function in humans is still controversial. It may be involved in the pathogenesis of insulin resistance [23]. Immunohistochemistry has shown that the gene expression of resistin is low in normal human liver but is increased in conditions of severe fibrosis [18]. Plasma resistin levels also increased, with a higher grade of liver damage in cirrhosis [24].

In patients with chronic HCV, the presence of steatosis can predict treatment failure (in the cases of pegylated interferon and ribavirin) [19, 20]. Compared with other predictors of treatment failure in HCV, such as genotype, viral load and ethnicity, the relationship between steatosis and treatment failure is less well understood. Studies have shown that IR might play a role in developing steatosis and treatment failure. It has recently been demonstrated that IR [measured by homoeostasis model assessment (HOMA), which is currently one of the most widely used indices based on fasting glucose and insulin] is an independent predictor of poor response to antiviral therapy in HCV patients [22]. Sustained virological response (SVR) in HCV genotype 1 patients and IR was around a half (32.8%), as compared with the SVR rate in HCV genotype 1 patients without IR (60.5 %) [22]; however, with the recent FDA-approved direct acting antiviral treatments and a high rate of SVR, steatosis might not play a significant role in treatment failure.

## Diagnosis

Currently, liver biopsy is the ‘gold standard’ for assessing the severity of hepatic fat deposition but biopsy is an invasive procedure and, in some patients, will result in complications such as internal bleeding, biliary leakage, hematoma formation, and infection. Up to 3% of patients require hospitalization after elective biopsy. The cost of biopsy is another important issue [5]: this is why attention has shifted to non-invasive measures of hepatic fat detection, using radiological modalities.

## Imaging of hepatic steatosis in chronic hepatitis C

Various imaging techniques are used for the detection of hepatic steatosis: -

### Ultrasound

Hepatic steatosis appears as a diffuse increase in hepatic echogenecity [25]. Other clues to the presence of fatty liver on ultrasound are (i) a more echogenic appearance of the liver parenchyma when compared with the adjacent right kidney and (ii) blurring of the internal structures of the liver, such as portal vein branches or biliary system [25]. Ultrasound is a widely available and inexpensive imaging modality that does not involve radiation. Its drawbacks include its inability to quantify the degree of steatosis and being operator-dependent [25, 26].

### Computed tomography

Hepatic steatosis in non-enhanced computed tomography (CT) images appears hypodense due to the inverse relationship between hepatic fat content and hepatic attenuation [27].

The difference between the attenuation values of the liver and spleen—which are measured in Hounsfield units (HU)—is used to diagnose hepatic steatosis. Healthy livers will have an attenuation value of 50–57 HU, 8–10 HU higher than that of the spleen [28]. A liver with attenuation values of less than 40 HU (or 10 HU less than spleen) is highly predictive of hepatic steatosis [29], but CT is more expensive than ultrasound and exposes the patient to radiation [30].

### Magnetic resonance imaging

Magnetic resonance imaging (MRI) techniques are being increasingly accepted as relatively quick, accurate, and non-invasive methods for detecting hepatic steatosis [31]. The most frequently-used technique for the detection of hepatic steatosis involves the in-phase and opposed-phase measurement methods, which exploit the frequency difference between water and lipid signals to generate in-phase and opposed-phase images [32]. In fatty liver, a drop in signal of the liver parenchyma is seen in the opposed-phase image when compared with the in-phase [31]. Using this technique, sensitivity and specificity for the detection of moderate-to-severe hepatic steatosis are greater than 80% and 95%, respectively [33]. In addition, MRI can be used for the quantification of fat content of the liver parenchyma [31].

## Diabetes

Hepatitis C has various extrahepatic immunologically mediated syndromes, including essential mixed cryoglobulinemia, porphyria cutanea tarda, membranous glomerulonephritis, Mooren cornea ulcer, Sjögren syndrome, autoimmune thyroiditis, lichen planus, and idiopathic pulmonary fibrosis. Diabetes mellitus (DM) is another important extrahepatic manifestation of hepatitis C [34–37]. It has been shown that DM and impaired glucose tolerance—which is a precursor of DM—have higher prevalence in hepatitis C [34, 37, 38]. As a matter of fact, the risk of DM is three times as high in chronic HCV as in those patients with non-C hepatitis [25]. A case control study by Knobler *et al.* showed that prevalence of DM is 33% in non-cirrhotic patients with chronic hepatitis C, compared with 5.6% in a control group [39].

It has been shown that patients with chronic hepatitis C have a high prevalence of glycometabolic abnormalities—such as glucose intolerance in more than 40% and DM in more than 17 % [40, 41]. The incidence of new-onset glucose abnormalities—including DM in HCV patients who achieved sustained virological response (SVR) after therapy—was lower than the incidence in those who failed to respond to therapy. This means that HCV should have a direct effect in the development of glucose intolerance and DM [42]. Another study has also shown that lower incidence of diabetes type 2 is expected in cured patients when compared with non-responders and this supports the notion of better control of insulin resistance after clearance of the hepatitis C virus [43].

As mentioned, HCV genotype plays a role in the occurrence of glucose metabolic disorders; genotypes 1 and 4 are significantly associated with IR more frequently than genotypes 2 and 3 (37% *vs.* 17%) [40].

DM presents an added risk in hepatitis C patients, due to rapid fibrosis progression compared with HCV patients without DM [39]. Also, the presence of DM in a patient with chronic HCV infection increases the risk of hepatocellular carcinoma (HCC) [44]. Ultimately patients with HCC and DM have a higher risk of mortality than patients with HCC without DM [41].

**Figure 3.**
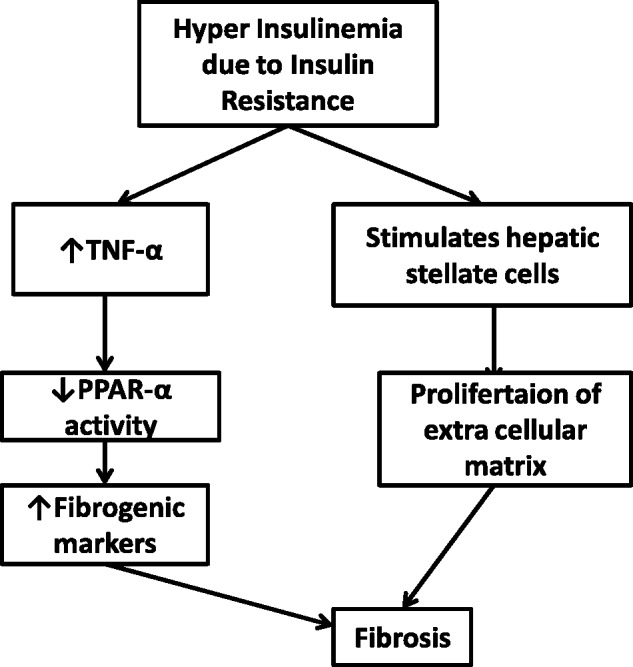
Hyperinsulinemia and liver fibrosis

Diabetic patients have hyperinsulinemia, which promotes and accelerates the progression of fibrosis in patients with HCV through several mechanisms, including hepatic steatosis, tumor necrosis factor alfa (TNFA) production, and impaired expression of PPAR-α [32]. PPAR-α plays a central role in regulating fatty acid transport and catabolism. Additionally, hyperinsulinemia may directly stimulate hepatic stellate cells to proliferate and secrete extra cellular matrix, leading to acceleration in fibrosis progression (Figure 3) [43].

It seems that HCV patients with DM may need to be treated with the novel direct antiviral agents (DDAs) as they are difficult to treat patients [45].

We still do not understand the precise mechanism by which DM negatively affects SVR in chronic hepatitis C patients; peginterferon induces their antiviral activity via extracellular receptor binding, which will cause activation of Janus kinase (Jak1) and tyrosine kinase (Tyk2). This leads to the activation of different substrates, signal transducers and activators of transcription such as (STAT 1) and (STAT 2). Finally, activated STAT will translocate into the nucleus as a complex. It has been shown that hyperinsulinemia and obesity interferes with the intracellular signaling of PEG-IFN alpha by activating the phosphatidil-inositol-3-kinase (PI3K) which blocks STAT-1 translocation, avoiding the antiviral effect of interferon. Also, elevated TNF-α levels are seen in subjects with DM and in HCV patients who failed to achieve SVR following therapy with interferon (IFN) [43, 45]. Hotamisligil *et al.* have shown that adipocytes constitutively express the pro-inflammatory cytokine TNF-α and that TNF-α expression in the adipocytes of obese animals is markedly increased [46]. Because of the inhibitory effect of TNF-α on IFN-α intracellular signaling, a recent randomized clinical trial suggested that neutralization of TNF-α by a soluble TNF-α receptor improves the response to non-PEG-IFN/RBV therapy in HCV patients [47].

## Obesity

Of the several metabolic abnormalities associated with HCV, obesity is of particular interest due to its increasing prevalence among adults and children and for its potentially preventable and modifiable nature. Obesity has reached epidemic proportions in the United States [48]. More than one-third of adults and 17% of young people in the United States are obese [48]. Obesity also affects every segment of the US population and continues to increase steadily, especially in children [48].

Chronic hepatitis C patients who are obese are at greater risk of HCC [49]. Another study has shown that obesity itself—unrelated to infections with HCV and/or HBV, drinking and smoking habits—increases the risk of HCC [50, 51].

Women with a BMI of ≥35 kg/m^2^ had a relative risk of death from HCC of 4.52 (95% CI: 2.94–9.94) compared with those with lower BMI [51]. Women with BMI ≥35 kg/m^2^ were also at higher risk of death from HCC than women with lower BMI, although the risk was less significant than in men (relative risk 1.68) [51]. A more recent and larger study utilizing the UNOS (United Network for Organ Sharing) database of patients who underwent Orthotopic Liver Transplantation (OLT) in the USA also suggested that BMI is an independent risk factor for the development of HCC in patients with cirrhosis due to alcoholic or cryptogenic liver disease [52].

Liver steatosis has been reported in up to 60% of obese subjects [14]. An association between liver steatosis and HCC has recently been reported among HCV-positive patients [4]. It has been shown that, in healthy individuals, PPAR-α acts to ameliorate steatosis but, in the presence of mitochondrial dysfunction—which can be seen in HCV patients—PPAR-α may exacerbate steatosis [8]. Lipid peroxidation, which induces mutagens from reactive oxygen species, is the possible reason for the development of cancer-promoting mutations [53]. In addition, both obesity and diabetes were associated with insulin resistance and elevated insulin-like growth factor, which stimulate cell proliferation, cell differentiation and potent inhibition of apoptosis. These ultimately may the promote proliferation of cancer cells [54].

Obesity—often associated with IR—is independently associated with advanced stage fibrosis, supporting an intimate interplay between metabolic factors and liver injury [45].

It has been shown that weight loss can improve liver biochemistry profiles; a loss of at least 10% of body mass in 39 obese patients was associated with reversal of abnormal liver function tests, as well as decreased hepatomegaly [55].

Obesity negatively affects treatment outcomes [56]. The SVR rate in patients with HCV genotype 1 infection, who are treated with PEG-IFN and ribavirin, is significantly lower in those with IR than in those without it [22].

## Metabolic syndrome

Metabolic syndrome (MS)—characterized by a constellation of factors including obesity, impaired fasting glucose, hypertension, increased waist circumference and dyslipidemia—has been a major public health problem in the United States and many other parts of the world. A growing body of evidence suggests that several components of MS may be important co-factors in chronic HCV-infected patients [3, 4]. Obesity and IR are strong risk factors for MS. Increased waist circumference (excess of abdominal body fat) in obese patients is deleterious, whereas peripheral body fat is neutral or even protective against metabolic abnormalities [57].

MS is now considered to be a clinical entity associated with the presence of IR at the cellular level and with endothelial dysfunction, and its related clinically adverse cardiovascular outcome. The association of pro-inflammatory cytokines with MS is well established. Interleukin-6 (IL-6), resistin and TNF-α are overproduced by the expanded adipose tissue mass and the excessive number of monocyte-derived macrophages. Together with a chronic, low-grade inflammatory state, these factors induce insulin resistin in adipose, liver and muscle tissues. On the other hand, adiponectin, an anti-inflammatory cytokine secreted mainly by adipocytes, may also be involved. This substance enhances insulin sensitivity and inhibits several steps of the inflammatory pathway. Low adiponectin levels have been shown to be consistently associated with NASH, which is another common feature of MS [57].

Studies have shown that HCV genotype has an effect on the prevalence of MS. The higher prevalence of MS in patients with HCV genotype 1 infection, compared with those infected with a non-1 genotype, is interesting and may suggest a specific role of the HCV genotype 1 virus in inducing metabolic abnormalities such as IR [45].

There is a strong association between MS and treatment outcomes (using PEG-IFN and ribavirin) in patients with chronic hepatitis C. MS was significantly associated with the lack of SVR after adjusting for ethnicity, genotype, obesity, baseline HCV RNA, steatosis and fibrosis. Subjects with MS were 3.8 times more likely to fail to respond to treatment than those without MS [45].

*Conflict of interest statement*: none declared.
